# P-434. Comparison of Cardiovascular Disease Risk Calculators in People Living with HIV in the US Military Natural History Study

**DOI:** 10.1093/ofid/ofae631.634

**Published:** 2025-01-29

**Authors:** Caitlin Bettger, Mackensie Horn, James Aden, Xiaohe Xu, Brian Agan, Jason Blaylock, Derek Larson, Robert O’Connell, Rhonda Colombo, Tahaniyat Lalani, Joseph Yabes, Ana E Markelz

**Affiliations:** San Antonio Uniformed Services Health Education Consortium, Fort Sam Houston, Texas; Infectious Disease Clinical Research Program, Bethesda, Maryland; BAMC, San Antonio, Texas; The University of Texas at San Antonio, San Antonio, Texas; Infectious Disease Clinical Research Program, Department of Preventive Medicine and Biostatistics, Uniformed Services University of the Health Sciences, Bethesda, MD, USA, Bethesda, Maryland; Walter Reed National Military Medical Center, Bethesda, Maryland; Fort Belvoir Community Hospital and Uniformed Services University, Fort Belvoir, Virginia; Infectious Disease Clinical Research Program, Bethesda, Maryland; Infectious Disease Clinical Research Program, Bethesda, Maryland; Infectious Disease Clinical Research Program, Bethesda, Maryland; Brooke Army Medical Center, San Antonio, Texas; Brooke Army Medical Center, San Antonio, Texas

## Abstract

**Background:**

Primary prevention of atherosclerotic cardiovascular disease (ASCVD) is a vital component of care for people living with HIV (PLWH). Current guidelines recommend at least moderate intensity statin for low to moderate ASCVD risk and high intensity statin for high risk. Determining ASCVD risk in PLWH is difficult due to a paucity of precise risk calculators. We retrospectively compared available ASCVD risk calculators in a population who had known ASCVD events.

**Figure:**
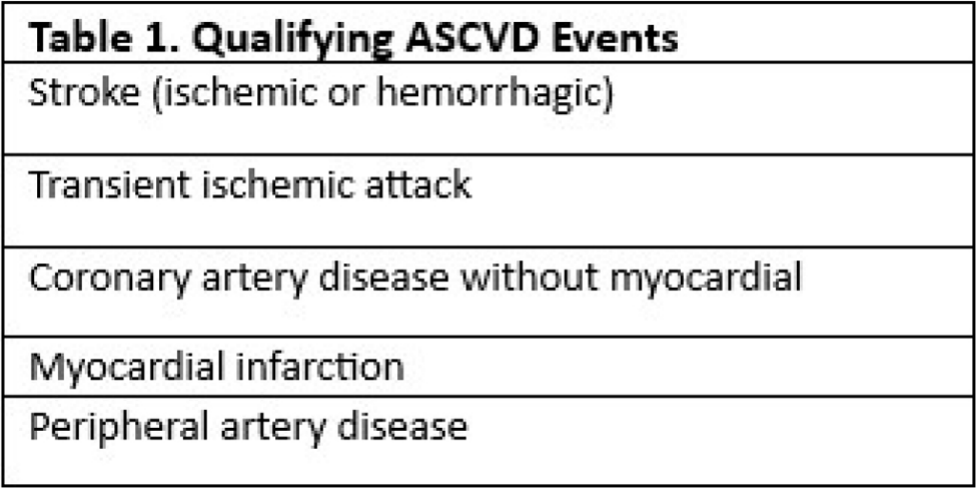

**Methods:**

We included US Military HIV Natural History Study (NHS) participants between 2006-2019 who experienced a known ASCVD event as defined in Table 1. Participants missing data to perform all four calculators were excluded from the analysis. Four risk calculators: American College of Cardiology/American Heart Association Pooled Cohort Equation (PCE), Framingham Heart Study Risk Score (FRS), Data Collection on Adverse Effects of Anti-HIV Drugs (D:A:D) model, and the newly published HIV-CARDIO-PREDICT (HCP) were applied for each participant at 10, 5, and 2 years prior to the ASCVD event. Proportion of participants categorized as high risk was determined for each calculator. Paired calculators were compared by Fisher’s exact or Chi-squared tests.

**Figure:**
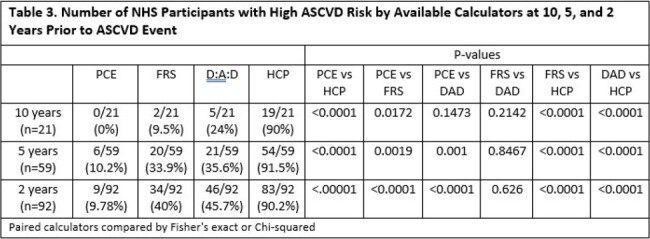

**Results:**

A total of 134 participants met initial inclusion criteria (Table 2). Of these, 113, 75, and 42 were excluded from the 10, 5, and 2 year analyses respectively due to missing data. HCP had the highest percentage of high-risk participants at any given time interval (Table 3). PCE had the lowest percentage of high-risk participants across all intervals. Head-to head comparison of PCE versus HCP demonstrated a significant difference in proportion of participants classified as high risk at all time intervals (p < 0.0001).

**Figure:**
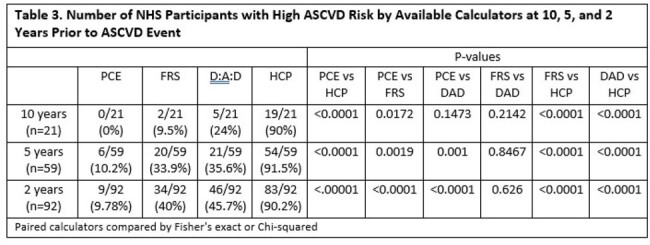

**Conclusion:**

In this military study cohort of PLWH who had a known ASCVD event, HCP had the highest sensitivity in classifying participants as high risk at all intervals. PCE, which is guideline recommended for ASCVD risk analysis, had the worst sensitivity. Further understanding of these calculator scores including their driving components over time (especially for HCP), their correlations with outcomes when non-cases are included, and their predictive ability among younger participants are needed.

**Disclosures:**

**All Authors**: No reported disclosures

